# Caregiver Burden in Movement Disorders and Neurodegenerative Diseases: Editorial

**DOI:** 10.3390/brainsci12091184

**Published:** 2022-09-02

**Authors:** Martin Klietz

**Affiliations:** Department of Neurology, Hannover Medical School, Carl-Neuberg-Straße 1, 30625 Hannover, Germany; klietz.martin@mh-hannover.de; Tel.: +49-511-532-3122; Fax: +49-511-532-8110

Caregiver burden is still an unmet need in the treatment of many neurodegenerative diseases. In movement and neurodegenerative disorders, the patient often experiences a progressive loss of autonomy and reduced quality of life [1]. The patient needs the help of an informal caregiver to cope with the activities of daily living [2]. These caregivers experience a multifactorial burden, which can be referred to as caregiver burden [3]. Besides the huge socioeconomic influence, caregiver burden is an orphan topic in neurodegenerative and movement disorders [4,5,6]. Hence, with progressive burden, the caregiver suffers from depressive mood and reduced quality of life [7]. Finally, in the case of caregiver burnout, the informal caregiver cannot further support the patient, and this often leads to institutionalization of the patient [8]. Strategies for the relief of caregiver burden and the prevention of caregiver burnout are desperately needed.

This Special Issue was conducted to stimulate diverse research projects in this scientific area and close current gaps of knowledge. Several experts in the field of Parkinson’s disease (PD) and atypical Parkinsonism, Dementia, amyotrophic lateral sclerosis, and Huntington’s disease (HD) contributed to this Special Issue. Overall, this Special Issue includes 17 articles focusing on different diseases and aspects of caregiver burden. We hope that this Special Issue raised awareness and stimulated the scientific process to finally develop and improve specific therapies for caregiver burden in the future.

In the area of Parkinson’s disease, Klietz et al. presented data on the longitudinal trajectory of caregiver burden in a longitudinal questionnaire-based study [9]. In this study, a disease specific questionnaire for the assessment of PD caregiver burden was used [10,11]. Overall, the authors reported no significant changes in caregiver burden over the time of one year, also no gender-specific differences were detected in the caregiver task questionnaire.

Gülke et al. discussed different aspects of burden in PD caregivers under deep brain stimulation in a very informative narrative review based on a structured screening of the literature [12]. The authors focus on perioperative aspects influencing caregiver burden in these patients and their spouses.

In a secondary data analysis of a large real-world cross-sectional cohort, Kinateder et al. analyzed the prevalence of sexual dysfunction in PD and the impact on the relationship [13]. Whereas male PD patients most frequently complained of erectile dysfunction, female patients complained in the majority about orgasm dysfunction and reduced libido. These reported symptoms were associated with negative effects on the sexual relationship.

Data on relationship satisfaction in PD patients and their spouses were analyzed in a cross-sectional questionnaire-based study by Heine et al. [14]. The authors reported that reduced relationship satisfaction in PD caregivers was associated with reduced quality of life, caregiver burden, depressive mood, and other neuropsychiatric symptoms.

Eichel et al. also analyzed neuropsychiatric symptoms of PD patients in a cross-sectional questionnaire-based study [15]. In a multiple linear regression analysis reduced mood and apathy measured by the Scale for Evaluation of Neuropsychiatric Disorders in Parkinson’s Disease contribute to the burden of the caregiver.

In the study of Deutsch et al. semi-structured interviews were used to increase the understanding of the impact of delusions of PD patients on caregiver burden [16]. The authors were able to identify four main themes emerging from the caregivers experiencing delusions of PD patients prior to the interviews. The authors named these four themes “managing incredulity”, “hypervigilance”, “defensive strategies” and “concealing and exposing”. In conclusion, patient and caregiver education should be included in clinical care and clinicians should be aware of the impact of delusions on PD patients and caregivers.

By using a cross-sectional monocentric exploratory study, Vatter et al. investigated resilience in people with PD dementia and their caregivers [17]. Lower resilience was associated with higher anxiety and lower quality of life in patients with PD dementia. Further, caregivers with lower resilience reported reduced well-being, relationship satisfaction, and higher burden.

In a sub-study of the international prospective multicenter study “care for late-stage Parkinsonism” Rosqvist et al. analyzed the burden of the caregiver and its influence [18]. In a multivariant linear regression analysis, good cognitive function and female gender of the patient were associated with better quality of life of the caregiver. This study also pronounces the importance of identification and treatment of relevant non-motor symptoms in the advanced and late stages of PD [19].

Jensen et al. published a retrospective semiquantitative pilot study on the transition of PD patients to institutional care [8]. This study analyzed the decision process, the symptoms of the PD patient, the burden of the caregiver, and also caregiver tasks. The authors found that transition into an institutional care facility mainly elapsed in the advanced or late stages of PD, the decision was often delayed by months or even years. However, after transitioning into institutional care the symptoms of the PD patient remained stable contrary to a profound reduction in the burden of the caregiver. More research is needed in this area to develop treatment strategies and maintain patients’ autonomy while preventing excessive caregiver burden.

In this context, Muente et al. presented a study protocol for an e-health intervention to support relatives of PD patients prior to caregiver burden [20]. This interventional study aims to prevent caregiver burden on relatives of PD patients.

Thieken et al. discussed the caregiver of PD patients as an “invisible patient” in their opinion paper [21]. The authors discuss the development of caregiver burden and the trajectory of PD in a very comprehensive way and conclude that holistic approaches will be needed in future care to also address the needs of the caregiver in the complex treatment of PD patients.

In the second section of the Special Issue, two exciting studies on caregiver burden in Huntington’s disease have been contributed.

In the first study Exuzides et al. reported data from a large cross-sectional survey in the United States [22]. The authors reported that individuals with HD had a lower quality of life and more depressive symptoms. Care partners of individuals with HD only reported higher depression scores compared to the general population. Interestingly, the authors found no differences in individuals with HD and their care partners compared to individuals with PD and their care partners. This study highlights that besides some specific aspects of each disease there is an underlying general mechanism of caregiver burden.

Aschenbach et al. presented a cross-sectional study of the global enrollment HD registry [23]. In this huge cohort, the authors compared HD patients, premanifest mutation carriers, HD negative family members, and genetic negative control participants. The authors reported that family members of HD patients suffered from higher self-reported depression and lower cognitive function. The study draws attention to the complex situation of HD family caregivers.

In the field of dementia, Schaffler-Schaden et al. presented an exploration of caregiver of people with dementia living in rural areas [24]. In this survey, the experience of caregiver burden in women and men differed significantly. Factors contributing to the caregiver burden were behavioral aspects of the person with dementia and cohabitation with the caregiver. These results frame the need for tailored interventions for caregivers.

In the section on amyotrophic lateral sclerosis, three diverse and stimulating paper have been published with different methods and study samples.

Conroy et al. reported an analysis of an international exploratory study on the burden and difficulties of informal caregivers of ALS patients [25]. In this study, the most relevant difficulties were emotional factors associated with decreasing health status of the ALS patient, changes in the partnership, and self-experienced distress. The authors conclude, that more evidence for psychological support of these informal caregivers is needed.

Caregiver burden and negative consequences on the lives of informal caregivers of ALS patients were examined by Schischlevskij et al. [26]. In this data analysis of the German motor neuron disease network, 249 informal caregivers were included in this evaluation. The main predictors of caregiver burden in this study were increasing disease severity and loss of autonomy of the ALS patient.

In the mixed methods pilot study on the role of thick mucus in ALS patients, Bublitz et al. conducted semi-structured interviews [27]. Patients with thick mucus reported lower quality of life as well as their caregivers. This study raises awareness, that some specific symptoms of a disease may contribute to a large proportion of caregiver distress and burden.

This Special Issue presents a diverse overview of the field of caregiver burden in movement disorders and neurodegenerative diseases. A huge variety of different topics has been addressed. This Special Issue is of great interest to neurologists, psychologists, general physicians, and nursing scientists.
